# Specific recruitment properties of spinal reflex of thigh muscle in sprinter

**DOI:** 10.1038/s41598-025-22504-2

**Published:** 2025-11-04

**Authors:** Gaku Kakehata, Kento Nakagawa, Naotsugu Kaneko, Yohei Masugi, Shigeo Iso, Kimitaka Nakazawa

**Affiliations:** 1 Faculty of Sport Sciences, Waseda University, Tokorozawa, Saitama Japan; 2 Graduate School of Arts and Sciences, The University of Tokyo, Meguro-Ku, Tokyo Japan; 3 Japan Society for the Promotion of Science, Chiyoda-Ku, Tokyo Japan; 4 Department of Sports and Health Management, Jobu University, 634-1 Toyazukamachi, , Isesaki-Shi, Gunma 372-8588 Japan; 5 Department of Physical Therapy, School of Health Sciences, Tokyo International University, Kawagoe, Saitama Japan

**Keywords:** Motor control, Neuronal physiology

## Abstract

Thigh muscles activation pattern plays a key role in high-performance sprint running. We investigated spinal neural mechanisms underlying sprinters’ specific thigh muscle control by examining spinal reflex recruitment properties and reciprocal inhibition of the thigh muscles in sprinters. Ten high-level national sprinters and ten controls participated in this study. Transcutaneous spinal cord stimulation (tSCS) was used to evoke spinal reflexes in biceps femoris (BF) and soleus (SOL) muscles. To assess the recruitment properties of thigh muscles, in Experiment 1, tSCS was applied at varying intensities to analyze recruitment curves. In Experiments 2–4, reciprocal inhibition of the thigh muscles was assessed under electrical stimulation, voluntary contraction, and muscle vibration. Sprinters exhibited a significantly steeper recruitment curve slope for BF spinal reflex compared to controls, whereas the groups did not differ for the SOL. Reciprocal inhibition from the rectus femoris to BF showed no significant differences between groups. These findings suggest that spinal neural circuits in sprinters undergo plastic changes, allowing higher motor output induced by reflexes with small changes in sensory input. This sprinter-specific spinal reflex recruitment, particularly in BF, likely aligns with their specific thigh muscle activation patterns.

**Supplementary Information:**

The online version contains supplementary material available at 10.1038/s41598-025-22504-2.

## Introduction

Sprint running is a fundamental activity of various sports. Superior sprinters have been reported to exhibit rapid and greater thigh movements than recreational athletes^1^. In particular, sprinters showed greater angular velocity in the thigh, which is associated with running speed^1^. These movements result from thigh muscle activity. The thigh muscles play an important role in rapidly accomplishing the forward and backward swings of the leg during sprint running^2^. Sprinters with greater step frequency show clear switching between activation of rectus femoris (RF) and biceps femoris (BF) and less co-contraction of these muscles during the late-swing phase of sprinting^3^. In other words, superior sprinters have a greater ability to activate and relax their thigh muscles with proper timing. Further, both RF and BF muscles activities are significantly increased during high-speed sprinting than during moderate- or slow-speed running^2^ because there is a strategy to increase the mechanical work produced by the thigh muscles in the swing phase during high-speed sprinting to achieve rapid leg movements^4,5^.

Theoretical frameworks in motor control propose that skilled movement arises from plastic adaptations within central nervous system^6,7^. In particular, spinal reflex pathways are considered modifiable through training^8^ and may contribute to the efficiency and precision of motor output in skilled athletes, as these pathways can be finely tuned through training to support efficient and rapid motor output^9–11^. Investigating such reflex mechanisms in expert sprinters may therefore provide valuable insights into how spinal neural control contributes to high-level athletic performance. Although the characteristics of muscle activity of sprinters have been extensively studied, the neural mechanisms underlying their sophisticated skills in sprint running remain poorly understood. Sprinting requires explosive and rapid muscular output, which depends not only on the muscular system but also on finely tuned neural control mechanisms. In particular, spinal-level input–output properties, such as recruitment gain, and adjustment mechanisms via interneurons (e.g., reciprocal inhibition) may play a key role in enabling the precise and rapid muscle coordination required for sprint performance.

Numerous studies have shown that general athletes exhibit plastic changes in motor-related neural circuits, such as the primary motor area (M1) and spinal cord. In athletes, these changes are prominent in the neural circuits innervating the key muscles in sports or training^11–16^. That is, muscle-dependent neural plasticity has been observed in athletes. It is estimated that neural circuits innervating thigh muscles are plastically changed to promote sprinter-specific thigh muscle activities because thigh muscles are representative of sprinters in terms of muscle activity and structure^3,17^. Gait behavior is thought to be primarily controlled by a spinal central pattern generator (CPG)^18,19^. As spinal neural circuits have been considered to contribute to high-speed locomotion as well as low-speed walking, at least for animals^20,21^, human sprinters are also hypothesized to show plastic changes in spinal neural circuits that innervate the thigh muscles, rather than cortical neural circuits. As potential spinal neural mechanisms underlying the rapid switching of agonist and antagonist thigh muscle activations in sprinters, the two possibilities can be considered. First, the changes of thigh muscle reflex activation in response to afferent input, known as reflex recruitment gain, may be greater in sprinter. This gain, which reflects how steeply reflex responses increase with increasing input signal, is considered a key parameter in spinal sensorimotor control. Previous research has shown that strength training can lead to a steeper recruitment gain in lower leg muscles^9^, and sprinters typically engage in high-intensity strength training. Since rapid activation and deactivation of thigh muscles are essential for high-speed sprint running, the spinal reflex pathways innervating these muscles may undergo plastic adaptations that enhance recruitment gain, thereby supporting swift force production and muscle relaxation during running. Second, the reciprocal inhibition between the agonist and antagonist thigh muscles may be stronger in sprinters with superior locomotor function and reciprocal thigh muscle activation patterns^3^. This is supported by previous findings showing that reciprocal inhibition is associated with locomotor function; for example, patients with impaired locomotor function exhibit weak or absent reciprocal inhibition in the lower leg muscles^22^.

Spinal motor-related neural circuits have been widely evaluated using the Hoffmann-reflex (H-reflex). The H-reflex can be evoked by the electrical stimulation of Ia-sensory fibers in the peripheral nerve trunk. The H-reflex amplitude reflects monosynaptic spinal reflex excitability^23^. However, owing to technical limitations, the H-reflex can be measured only from a limited number of muscles, such as soleus (SOL)^23^, making it almost impossible to record the H-reflex from thigh muscles, especially the hamstrings. Conversely, a recently developed technique, transcutaneous spinal cord stimulation (tSCS) has been used as a reliable tool to simultaneously evaluate spinal reflex excitability from multiple muscles, including the thigh muscles ^24–26^. Stimulation via tSCS has been used to examine the properties of spinal reflex excitability in thigh and lower leg muscles because the spinal reflex elicited by tSCS has characteristics that are similar to those of the H-reflex^27–29^. Further, we have recently developed methods to evaluate 1) the recruitment properties of the spinal reflex induced by tSCS based on recruitment curve analysis^26^ and 2) the reciprocal inhibition of thigh muscles using a combination of tSCS and conditioning of the antagonist muscle^30^.

**Figure 1.**
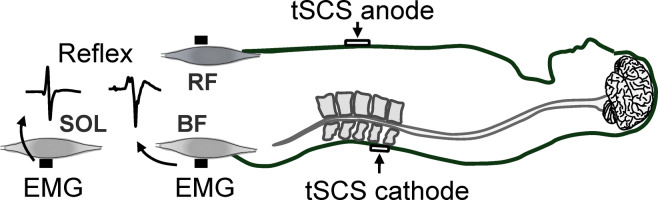
Experimental setup for the recording of the spinal reflex elicited by transcutaneous spinal cord stimulation (tSCS). Abbreviations: rectus femoris (RF), biceps femoris (BF), and soleus (SOL).

The objective of this study was to examine the properties of spinal neural circuits in high-level national sprinters. Our hypotheses were that 1) the recruitment gain of the spinal reflex of the thigh muscle is higher in sprinters compared to non-sprinters (controls) and that 2) the reciprocal inhibition between the thigh muscles is stronger in sprinters because superior sprint performance is associated with clear switching of the thigh muscle activations between RF and BF and minimum co-contraction of these muscles^3^. To test the first hypothesis, we evaluated the recruitment gain of the tSCS-evoked spinal reflex in BF muscle (Fig. 1). For the second hypothesis, we assessed the inhibition of tSCS-evoked BF spinal reflex by applying three types of Ia sensory nerve stimulation to the RF muscle. These measurements were then compared between sprinters and controls. 

## Results

### Experiment 1

The Boltzmann equation fit accounted for at least 95% of the total variance of the data points among all participants (R^2^ ≧ 0.95: mean ± SD, 0.986 ± 0.013 for BF in the Control, 0.991 ± 0.007 for BF in the Sprinter, 0.990 ± 0.011 for SOL in the Control, and 0.994 ± 0.007 for SOL in the Sprinter groups) (Supplementary Material 2).

**Figure 2.**
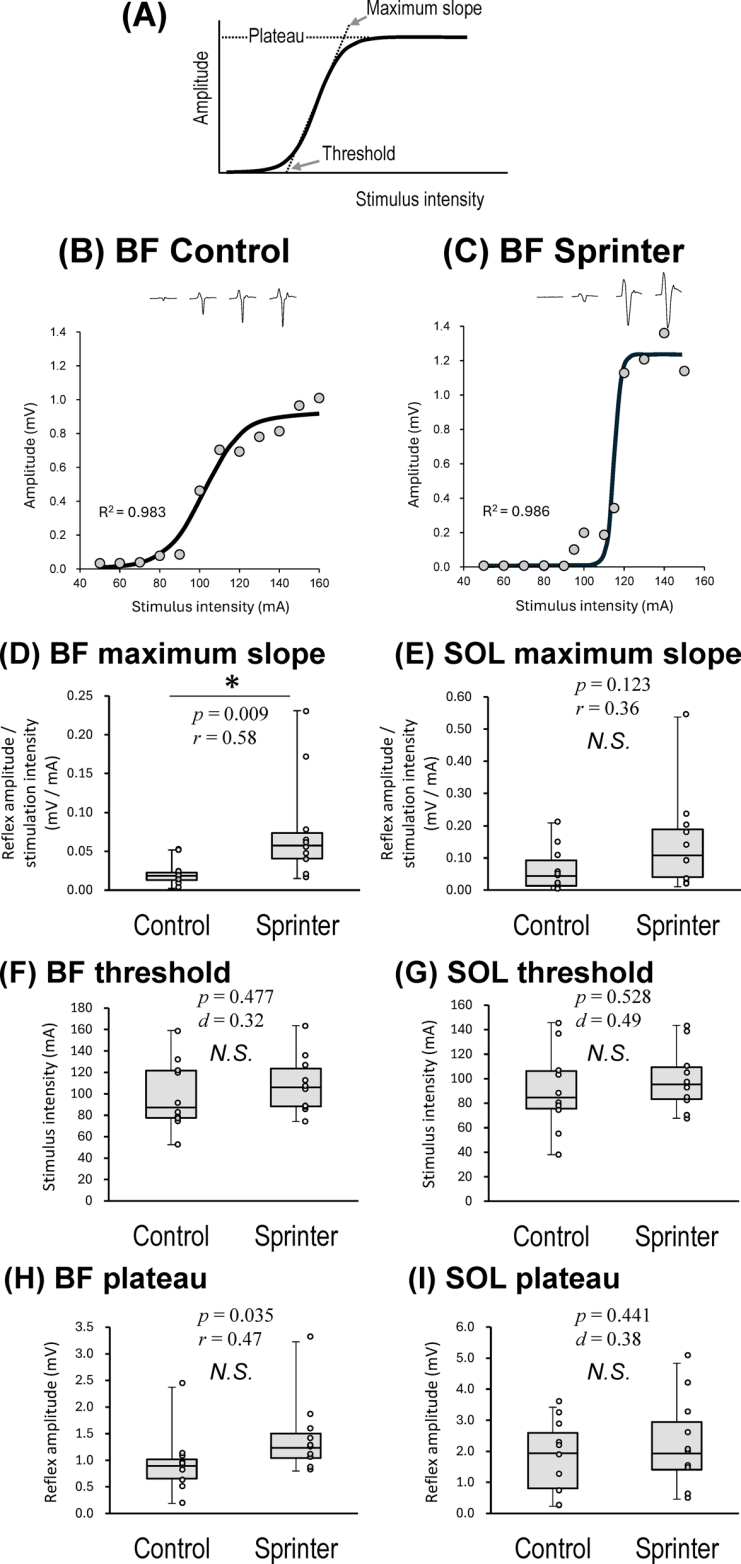
Recruitment properties of the spinal reflex in thigh muscles. (**A**) Schema for the calculation of each property in the recruitment curve of the spinal reflex. Representative examples are shown of the quantitative relationship between the reflex amplitude in biceps femoris (BF) muscle and the stimulation intensity using the curve fitting results of the Boltzmann function for the Control (**B**) and Sprinter (**C**) groups. The R^2^ values of these fittings were 0.983 for the Control and 0.992 for the Sprinter group. (**D**–**G**) Group data of the calculated parameters using the Boltzmann function curve fitting. Individual data points (white circles) are presented in addition to the box-and-whisker plots. The asterisk indicates the significant difference between the Control and Sprinter groups. N.S. denotes no significant difference between the groups. The level of significance in these comparisons was adjusted as *p* < 0.025 by Bonferroni correction. *P*-value and the value of size effect (Cohen’s *d* for parametric data, *r* for non-parametric data) are shown in each comparison.

Figure 2B and 2C illustrate the representative relationship between stimulus intensity and amplitude of BF spinal reflex with a fitted sigmoid curve for one participant in each group and some recorded waveforms of BF spinal reflex above each plot. The maximum slope of the BF recruitment curve was significantly steeper for the Sprinter group (0.08 ± 0.07 mV/mA) compared with the Control group (0.02 ± 0.02 mV/mA; *p* = 0.009, *r* = 0.58) (Fig. 2D). For the SOL, the slope did not differ significantly between the group (Control: 0.07 ± 0.07 mV/mA, Sprinter: 0.16 ± 0.16 mV/mA; *p* = 0.123, *r* = 0.36) (Fig. 2E). There were no significant differences in the threshold intensity between the two groups for BF (Control: 99.12 ± 32.75 mA, Sprinter: 108.90 ± 27.31 mA, *p* = 0.477, *t*(18) = 0.84, *d* = 0.32) (Fig. 2F) nor SOL (Control: 84.81 ± 33.60 mA, Sprinter: 99.53 ± 25.93 mA, *p* = 0.528, *t*(18) = 0.64, *d* = 0.49) (Fig. 2G). The plateau amplitude also did not differ between the groups for BF (Control: 0.93 ± 0.58 mV, Sprinter: 1.41 ± 0.71 mV, *p* = 0.035, *r* = 0.47) (Fig. 2H) or SOL (Control: 1.72 ± 1.15 mV, Sprinter: 2.21 ± 1.42 mV, *p* = 0.441, *t*(18) = 0.79, *d* = 0.38) (Fig. 2I).

Based on the sensitivity analysis, the current sample size has sufficient power (80%) to detect large effects (e.g., *d* ≈ 1.1 or *r* ≈ 0.5), but is underpowered to detect small to medium effects (Supplementary Material 3). These thresholds provide a useful context when interpreting non-significant results.

### Comparison between the first and second response in the double-pulse paradigm

**Figure 3.**
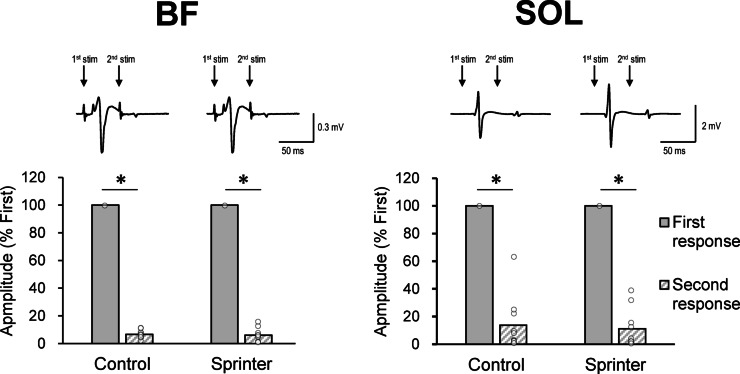
Amplitude of responses to the first and second stimulation in the double-pulse paradigm (50 ms interval) for the biceps femoris (BF: left) and soleus (SOL: right) muscles. The upper waveform represents the typical recordings of elicited responses from muscle evoked by double- pulse stimulation. The timings of the first and second stimulus are shown with black arrows. Note that the response after the second stimulus almost disappears for both muscles. The bar graph shows the quantified group data of mean amplitudes of the first and second responses. White circles represent individual data points. An asterisk indicates the significant difference in amplitude between the first and second responses.

Figure 3 shows a comparison of the amplitudes of the first and second responses at different stimulation intensities during Experiments 2–4. From the typical waveforms, the second response seemed to almost disappear, irrespective of the group or muscle. For both participant groups, the normalized amplitudes of the second responses were significantly smaller than those of the first response (100%) both for BF (Control: 6.57 ± 2.96%, *p* < 0.001, *t*(9) = 99.98,* d* = 44.71; Sprinter: 6.04 ± 4.83%, *p* < 0.001, *t*(9) = 61.51, *d* = 27.51) and SOL (Control: *p* < 0.001, *t*(9) = 14.18, *d* = 6.34; Sprinter: *p* < 0.001, *t*(9) = 20.51, *d* = 9.18). There were no significant differences in the normalized amplitudes of the second responses between the groups for BF (*p* = 0.770, *t*(18) = 0.30, *d* = 0.13) or SOL (*p* = 0.732, *t*(18) = 0.35, *d* = 0.16).

### Experiments 2–4

**Figure 4.**
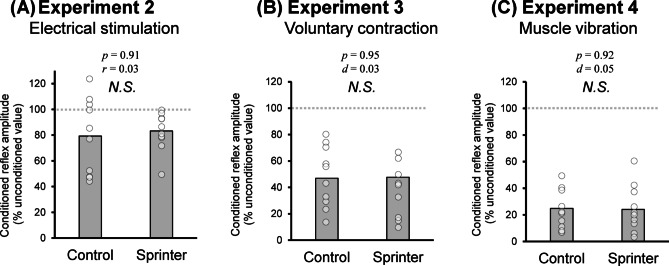
Comparisons of the reciprocal inhibition between the groups. Each bar indicates quantified group data of the amplitudes of the conditioned reflex in biceps femoris (BF) muscle in Experiments 2 (panel A), 3 (panel B) and 4 (panel C). White circles represent individual data points. A value of 100 in the vertical axis implies an unconditioned reflex amplitude. N.S. denotes no significant difference between groups. *P*-value and the value of size effect are shown in each comparison.

Regarding the stimulus intensity of tSCS used in Experiments 2–4, unpaired t-tests confirmed that there was no significant difference between groups (Control: 135.3 ± 38.0 mA, Sprinter: 142.3 ± 41.8 mA, *p* = 0.700). In Experiment 2, there was no significant difference in amplitude of BF reflex conditioned by the electrical stimulation on the femoral nerve between the two groups (Control: 79.37 ± 29.53% of the non-conditioned response, Sprinter: 83.29 ± 14.81% of the non-conditioned response, *p* = 0.912, *r* = 0.034) (Fig. 4A). In Experiment 3, there was no significant difference in amplitude of the conditioned reflex of BF during the voluntary contraction of RF between the groups (Control: 47.69 ± 22.87% of the non-conditioned response, Sprinter: 46.91 ± 36.22% of the non-conditioned response, *p* = 0.954, *t*(18) = 0.058, *d* = 0.03) (Fig. 4B). Normalized background EMG results during the voluntary contraction also did not significantly differ between the two groups for RF (Control: 11.91 ± 2.47%MVC, Sprinter: 11.11 ± 3.72%MVC, *p* = 0.581, *t*(18) = 0.56,* d* = 0.17) nor BF (Control: 3.48 ± 4.02%MVC, Sprinter: 2.32 ± 1.84%MVC, *p* = 0.420, *t*(18) = 0.83). In Experiment 4, there was no significant difference in amplitude of BF reflex conditioned by vibration on RF muscle between the groups (Control: 24.06 ± 14.70% of the unconditioned response, Sprinter: 24.82 ± 17.63% of the unconditioned response, *p* = 0.917, *t*(18) = 0.10, *d* = 0.05) (Fig. 4C). Normalized background EMG during RF vibration also did not significantly differ between the groups for RF (Control: 2.40 ± 0.89% MVC, Sprinter: 1.72 ± 0.80% MVC, *p* = 0.091, *t*(18) = 1.78, *d* = 0.79) nor BF (Control: 1.82 ± 1.05% MVC, Sprinter: 2.22 ± 1.75% MVC, *p* = 0.549, *t*(18) = 0.61, *d* = 0.27).

## Discussion

The objective of the present study was to investigate the properties of spinal neural circuits in high-level national sprinters. We evaluated spinal reflexes of thigh muscles specific to sprinters^3,17,31^. To confirm whether the tSCS-induced response was a spinal reflex, we preliminarily examined it using a double-pulse paradigm. When a double-pulse (50 ms interval) stimulation was delivered, the response evoked by the second stimulation disappeared or was strongly suppressed (Fig. 3), which is in agreement with previous studies^28,30,32–34^. The suppression of the second response would be due to post-activation depression^35^ implying that the tSCS activated the sensory fibers. Therefore, the first response that we evaluated was likely the spinal reflex generated from the Ia sensory fibers, and its amplitude probably reflected the monosynaptic spinal reflex excitability.

Our hypotheses in the present study were as follows: 1) the recruitment gain of the spinal reflex of the thigh muscle is higher in sprinters, and 2) the reciprocal inhibition between thigh muscles is stronger in sprinters. Regarding the first hypothesis, the results of Experiment 1 showed sprinter-specific and muscle-specific recruitment properties of the spinal reflex, namely, higher recruitment gain of the spinal reflex in BF but not prominent in SOL in the Sprinter group compared with the Control group (Fig. 2D and 2E). For the peak slope of the recruitment curve in BF muscle, a significant group difference was observed (*p* = 0.009), accompanied by a large effect size (*r* = 0.58). These findings indicate a robust difference in the spinal reflex properties of BF muscle between sprinters and controls. In contrast, the peak slope in SOL muscle did not reach statistical significance (*p* = 0.123), despite a moderate effect size (*r* = 0.36), which might be partly attributable to limited sensitivity due to the current sample size. This result should therefore be interpreted with caution, and future studies with larger sample sizes are needed to confirm or refute a potential difference in SOL reflex characteristics. As the plateau reflex amplitude of the recruitment curve for BF did not significantly differ between the groups (Fig. 2H and 2I), the observed higher recruitment gain in the Sprinter group was likely due to changes in the recruitment property of the spinal neural circuit caused by long-term sprint training rather than the difference in the reflex amplitude. However, careful caution should be required in interpreting these results because the effect size of the group difference in the plateau reflex amplitude for the BF was medium (*r* = 0.47). A higher recruitment gain indicates that a higher motor output induced by the reflex can be delivered with small changes in the sensory input. That is, by using this plastically changed spinal reflex circuit, high-level sprinters would be able to accomplish rapid recruitment of hamstring muscles and their sudden deactivation. This would be a reasonable explanation for the neural mechanisms of smooth switching between RF and BF activity in superior sprinters^3^. In addition, as explained above, compared to BF muscle, the group effect in the recruitment properties of the spinal reflex in SOL muscle was likely lower even considering lower statistical power (Fig. 2E), suggesting that at least the recruitment property of BF reflex would be more sprinter-specific compared to that of SOL reflex. This finding may be another explanation for the specific spinal neural circuit characteristics of sprinters. Ankle kinematics generated by the lower leg muscles, including SOL, are not explanatory variables of sprint running performance, but thigh and knee kinematics are associated with sprint running performance^36^. Our group has also shown greater muscle activation of BF, but not SOL during sprint running in sprinters compared to distance runners (under review). Therefore, SOL muscle is unlikely to have unique functional characteristics in sprinters. To sum up, the prominent sprinter-specific recruitment property prominent in BF that was observed in the present study likely reflects a characteristic of high-level sprinters who have a greater ability to control their thigh muscles^3^. Thus, it is reasonable to infer that plastic changes in the recruitment properties of spinal reflexes in specific muscles reflect the type of sport or training.

A steeper slope of the spinal reflex recruitment curve indicates an increase in the efficacy of the afferent volley through a decrease in presynaptic inhibition^37^. Thus, the sprinter-specific recruitment pattern of BF reflex may be due to presynaptic circuits. As an elevated sensitivity of the muscle spindle after explosive muscle contraction has been suggested in power athletes, including sprinters^38^, presynaptic efficiency associated with an increase in the sensitivity of BF muscle spindle at a specific input range may be plastically altered in sprinters who train daily with explosive muscle contraction of BF. Moreover, a previous study demonstrated a higher H-reflex recruitment gain in SOL during lengthening contractions of the antagonist tibialis anterior muscle compared with shortening contractions^39^. It is speculated that the antagonist BF muscle to the quadriceps induces plastic changes to increase recruitment gain through long-term sprint training because sprinters frequently conduct strong lengthening contractions of the quadriceps muscles during the contact phase of sprint running^40^.

In contrast to their support for the first hypothesis, our results from Experiments 2–4 failed to support the second hypothesis. That is, although we succeeded in evaluating reciprocal inhibition in BF muscles with the three interventions for RF muscle (i.e., inhibition of the conditioned reflex amplitude by the interventions), the degree of reciprocal inhibition did not significantly differ between the Sprinter and Control groups, irrespective of the conditioning types involved (Fig. 4). As explained above, sprinters exhibited the higher recruitment gain of BF spinal reflex, which may thereby produce the sprinter-specific thigh muscle activity without any specific spinal reciprocal inhibitory function. A possible alternative explanation for the lack of sprinter-specific reciprocal inhibition in the thigh muscles may be the experimental condition in the resting state or the weak contraction of RF muscle. According to a previous study that measured reciprocal inhibition of the lower leg during walking, such an inhibition is modulated depending on the walking phase^41^, which would help perform the desired gait movements smoothly. A previous study showed that wrestlers modulate the stretch reflex of their upper limbs, which is important for a wrestling match in a task-dependent manner^42^. They also showed no difference in the stretch reflex amplitude between the wrestler and control groups in the resting state. Thus, some spinal neural circuits may only show training-dependent plastic changes during behaviors specific to the sport or training. Regarding the underlying mechanisms of task-dependent modulation, input from motor areas such as the M1 and the supplementary motor area (SMA) may play a role. Reciprocal inhibition has been reported to be enhanced by activity in both M1^43^ and SMA^44^. In this context, sprint running, being a complex motor task, is likely to engage these cortical regions. Notably, athletes have been shown to exhibit plastic changes in M1^13^ and SMA^45,46^, suggesting that descending input from these areas may facilitate reciprocal inhibition during sport-specific movements. By contrast, the present study involved isolated muscle contractions and passive afferent stimulation, which likely required minimal engagement of higher-order motor regions. This may partly explain the absence of group differences in reciprocal inhibition observed in our experimental paradigm. Furthermore, although 10% MVC was selected to isolate low-threshold intermuscular reflex responses and ensure signal stability, we acknowledge that this level of contraction may not capture the full scope of reflex modulation that occurs during high-intensity, performance-relevant activities such as sprinting. Given the known differences in muscle recruitment strategies and cortical involvement during explosive movements, future studies incorporating higher contraction intensities or more task-specific protocols may provide further insights into the functional relevance of the observed neural differences. Specifically, sprinter-specific reciprocal inhibition of thigh muscles, if any, may appear during dynamic running movements involving strong intensity, where supraspinal drive is substantially elevated, but not in a resting state or during weak contraction, as in the present study.

The present study demonstrated the possibility of plastic changes in the spinal neural circuits innervating the thigh muscles in high-level sprinters. This finding suggests a possible association between spinal neural function and sprint running performance. From this perspective, the causal relationship between them should be investigated using noninvasive spinal cord stimulation techniques. At lower walking speeds, transcutaneous electrical or magnetic stimulation of the spinal cord can promote walking function in patients with lower limb paralysis^47,48^. In addition, in the sprint cycling exercise, which is required alternative activations of the quadriceps and hamstrings muscles like sprint running^49^, transspinal direct current stimulation on the lumbar spinal cord has been shown to facilitate sprint cycling performance^50^. Therefore, these neuromodulation techniques may be applicable to sprint running to improve sprint performance by using stimulus parameters that increase the recruitment gain of the thigh muscle reflex. This may lead to the development of new training methods that involve the central nervous system in sprint running.

When interpreting non-significant results, it is important to consider the statistical sensitivity of the sample. Our sensitivity analysis indicates that the current sample size was sufficient to detect large effects, but may have lacked power to detect more moderate effects. This limitation should be considered in future studies aiming to replicate or extend these findings. Limitations of the current methodology should also be considered. In some participants, the reflex recruitment curve did not reach a distinct plateau (Supplementary Material 2) due to the occurrence of a second response exceeding 0.1 mV. To avoid contamination by non-reflex components such as direct motor response, the stimulation intensity was not further increased beyond this point. Although the curve fitting still showed high R^2^ values (≥ 0.95), the absence of data near the plateau may have compromised the accuracy of the plateau estimation and thus affected the determination of the *S*_*50*_ intensity. This limitation should be taken into account in future studies aiming to replicate or refine the current experimental protocol.

In conclusion, the present study demonstrated that high-level national sprinters show specific recruitment properties of the spinal reflex, namely, a larger gain of reflex recruitment, especially in BF muscle, which is likely one of the neural mechanisms of sprinter-specific control of the thigh muscles. Additionally, reciprocal inhibition of the thigh muscles in the sprinters did not differ from that in the control participants in the experimental setting of the present study.

## Methods

### Participants

Ten high-level national sprinters (Sprinter group, personal best record of 100 m sprint: 10.51 ± 0.17 s; age, 20 ± 2-years; height, 176 ± 6 cm; weight, 69 ± 6 kg) and ten control males (Control group, age, 22 ± 3 years; height, 175 ± 2 cm; weight, 65 ± 6 kg) were enrolled. Individuals in the Control group had no competitive experience as sprinters but were recreationally active and had no neurological disorders. Written informed consent was obtained from all participants. This study was approved by the Human Research Ethics Committee of Waseda University (Ethical Approval Number: 2021–092). This study was conducted in accordance with the principles of the Declaration of Helsinki. We did not recruit women because of methodological limitations (i.e., the electrodes were attached to the skin surface of the trunk and groin).

### Electromyography (EMG) recordings

Surface EMG signals were recorded at 2000-Hz using a Delsys Trigno EMG system (Delsys Inc., Natick, MA, USA). The signals were band-pass filtered between 10 and 850-Hz. EMG data were obtained from BF, RF, and SOL muscles. EMG sensors were placed on RF halfway along a line drawn from the anterior spina iliaca superior to the superior part of the patella and on BF halfway along a line drawn between the ischial tuberosity and the lateral epicondyle of the tibia^51^. Prior to the attachment of the sensors, the involved area of the skin was shaved and treated with alcohol to reduce interelectrode impedance. EMG signals from the two muscles were checked after placing the electrodes. The signals were transferred to an A/D converter (Power Lab; AD Instruments, Sydney, Australia) and stored on a computer. The researchers monitored EMG signals online. When an EMG signal was observed during the resting condition (Experiments 1 and 2), the researchers instructed the participants to relax and a tSCS stimulation was not initiated until the EMG signals had disappeared.

### Spinal reflexes evoked by tSCS

Figure 1 summarizes the tSCS paradigm. The participants were in a supine position with their knees fully extended, and both ankle joints were fixed by a strap to ankle–foot orthoses (Sakai-Seisakusyo, Tokyo, Japan) in the neutral position to ensure symmetrical positioning of the limbs. We recorded spinal reflexes from BF muscle with a constant-current electrical stimulator with a single 200-μs rectangular monophasic pulse (DS7AH, Digitimer Ltd., Welwyn Garden City, Hertfordshire, UK). A cathode (50-mm × 50-mm) was placed on the skin at the midline between the spinous processes, and an anode (100-mm × 75-mm) was placed on the midline of the abdomen between the xiphoid process of the sternum and umbilicus. The lower edge of the anode electrode was approximately 1-cm from the umbilicus. The cathode was placed where a single-pulse stimulation produced the largest response in BF and SOL muscles at the lower thoracic and upper lumbar vertebrae. The cathode was placed between L1 and L2 for all participants. Stimulation of L1 and L2 has been suggested to activate sensory fibers, whereas stimulation of more caudal vertebrae such as L5 or S1 induces direct motor root activation^52^. After an optimal placement of electrodes, we obtained the recruitment curves of the reflex amplitudes, as described in Experiment 1.

### Experiment 1: recruitment properties of the spinal reflex

A double-pulse stimulation paradigm (interpulse interval: 50-ms) was used to confirm that the evoked responses originated from sensory fibers, in accordance with previous studies^24,26,34^. The recruitment properties of the first and second responses evoked by tSCS were acquired to assess stimulus intensity for each participant. If the amplitude of the second response was greater than 0.1-mV, the trial was excluded from the calculation of recruitment properties^26^. This was done because the appearance of the second response indicated that the activation of motor fibers, rather than Ia-sensory fibers, was included in the first response. To record the recruitment curves, we gradually increased the stimulation intensity up to obtaining the maximum reflex amplitudes from BF and SOL muscles. The participants were asked to remain in a relaxed state.

All data points of the first response for which the amplitude of the second response did not exceed 0.1-mV were included for evaluation. To compare the input–output properties of spinal reflex recruitment between the groups in detail, the relationship between reflex amplitude and stimulus intensity was evaluated by fitting a Boltzmann sigmoidal curve for each participant^39,53,54^ (Fig. 2A). The Boltzmann equation relating the spinal reflex to the stimulus intensity (S) is described by the following equation:

$$Reflex\left(S\right)=\frac{{Reflex}_{max}}{1+e(\frac{{S}_{50}-S}{k})}$$

We quantified the three parameters of this function, namely the plateau value of the reflex amplitude (plateau), the stimulus intensity required to obtain a response of 50% of the plateau value (*S*_*50*_), and the slope parameter (*k*). The inverse of the slope parameter (1/*k*) is directly proportional to the maximal steepness of the function, which occurs at *S*_*50*_. We calculated the maximum slope directly by differentiating the input–output property equation and defined it as the gain^39^. The threshold, represented by the x-intercept, was calculated by fitting a tangential line to the point of maximum slope (threshold intensity)^55^. In addition, the reflex amplitude at the stimulus intensity required to reach a plateau value (plateau amplitude) was also calculated.

For the statistical analysis, we first assessed whether the Boltzmann-derived parameters (slope, threshold, and plateau) were normally and homogeneously distributed using the Shapiro–Wilk test and Levene’s test. As the BF plateau, the slope of both BF and SOL, were not normally distributed, these variables were analyzed using the Mann–Whitney U test to compare the Sprinter and Control groups. For datasets that met normality and homogeneity assumptions, unpaired t-tests were applied. Since each parameter was compared twice (i.e., for BF and SOL), the significance level was adjusted to *p* < 0.025 using Bonferroni correction in Experiment 1.

In addition to p-values, effect sizes (*d* and* r*) were calculated and interpreted according to standard thresholds: small (*d* = 0.2, *r* = 0.1), medium (*d* = 0.5, *r* = 0.3), and large (*d* ≥ 0.8, *r* ≥ 0.5) ^56,57^. Sensitivity power analysis was conducted using G*Power (version 3.1) to evaluate the minimum effect sizes that can be reliably detected with the current sample size (N = 10 per group, α = 0.05). This analysis followed the procedures outlined by Lakens ^58^. Power curves were generated across a range of effect sizes for both parametric (Cohen’s *d*, for t-test) and non-parametric (effect size *r*, for Mann–Whitney U test) tests (Supplementary Material 3).

### Setting the intensity of the test stimulation for experiments 2–4

Experiments 2–4 were conducted on the same day as Experiment 1. The intensity of the test stimulation that elicits the spinal reflex for Experiments 2–4 was set such that the amplitude of the first response was on the ascending part of the recruitment curve (approximately 70% of the plateau amplitude) ^30,59^. At this stimulus intensity, a large-amplitude response can be elicited, which is suitable for measuring the degree of inhibition (Experiments 2–4). Further, the set stimulus intensity can minimize the amplitude of the response induced by the second pulse in a double-pulse paradigm. Thus, our stimulus intensity in Experiments 2–4 was sufficient for observing reciprocal inhibition. The unpaired t-tests checked whether the stimulus intensity of tSCS differed between the group. Further, the amplitudes of the normalized second responses were compared between the groups via an unpaired t-test with Bonferroni correction.

After determining the stimulus location and intensity, we carried out Experiment 2, followed by Experiments 3 and 4. The study participants were allowed to rest ad libitum when they felt tired or sleepy.

### Experiment 2: reciprocal inhibition induced by electrical stimulation of femoral nerve

To compare the reciprocal inhibition within the thigh muscles between the Sprinter and Control groups, we conducted three types of stimulation interventions on RF muscle and assessed their inhibitory effects on BF spinal reflex. The methods used in Experiments 2–4 have been described in our previous study, which evaluated reciprocal inhibition within the thigh muscles ^30^.

In Experiment 2, reciprocal inhibition was assessed by evaluating changes in the amplitude of BF spinal reflex following conditioning electrical stimulation with a single pulse of 1-ms on the femoral nerve. Conditioning stimulation, generated by an electrical stimulator (SEN-7203; Nihon Kohden, Tokyo, Japan), activated the Ia-sensory fibers of RF. Using a pair of self-made electrodes, which consisted of a bolt embedded in a cork, we visually identified the site of conditioning stimulation where RF contracted without contractions of the other muscles, such as BF and adductor magnus. A visual inspection was performed instead of a quantitative EMG assessment because reliable quantification of BF muscle activity was not feasible. This limitation arose from large stimulation artifacts that obscured the early portions of the potential EMG signals. This issue is considered a methodological limitation of the present study. Stimulating electrodes with a 1-cm diameter for conditioning stimulation (inter-electrode interval: 2-cm) were placed on the skin surface of the groin over the femoral nerve trunk. Motor threshold (MT) was defined as the minimum stimulation intensity required to produce an RF muscle twitch that can be observed by visual inspection and palpation^60^. The intensity of the conditioning stimulation was set to 1.4-MT based on our previous study^30^. The inter-stimulus interval (ISI) for the conditioning reflexes was set at 10-ms (the tSCS was delivered 10-ms after the conditioning stimuli) because our previous study had demonstrated that a 10-ms ISI at 1.4-MT was the best conditioning to induce the strongest inhibition of BF spinal reflex^30^. ISI was controlled using a pulse controller (SEN-7203; Nihon Kohden, Tokyo, Japan).

Conditioned and unconditioned reflexes were recorded five times. Spinal reflexes evoked by test stimulation without conditioning stimulation (i.e., unconditioned reflex) were obtained prior to the conditioning test paradigms. For each participant, the conditioned reflex values were normalized to the unconditioned values. Throughout Experiment 1, participants were instructed to relax without any muscle contraction of the lower limbs.

To analyze spinal reflex excitability, the peak-to-peak amplitude within time windows of 5–45 ms after tSCS was calculated^61^. The mean values of the calculated amplitudes of conditioned and unconditioned reflexes were computed. Prior to statistical comparisons between the groups, normality and homogeneity of variance were assessed using the Shapiro–Wilk test and Levene’s test. In Experiment 2, the assumption of equal variances was violated; therefore, normalized conditioned reflex amplitudes were compared between the Sprinter and Control groups using the Mann–Whitney U test. In Experiment 3 and 4, normality and equal variance were confirmed, and unpaired t-tests were applied.

### Experiment 3: reciprocal inhibition induced by voluntary contraction of RF

The conditioning in Experiment 2 involved voluntary contraction of RF. The intensity of the voluntary contraction was 10% of the maximal voluntary contraction (MVC) that was conducted prior to task initiation. The participants were instructed to perform isometric hip flexion during the contraction and MVC tasks. Based on the EMG signal amplitudes of the MVC trials, we set the target line at 10% MVC, because higher contraction intensity may cause unintended co-contraction of antagonist muscles, and our previous study demonstrated that this level of contraction effectively induces reciprocal inhibition in the thigh muscles^30^. The participants were provided verbal feedback on the degree of muscle contraction in RF. The tSCS was delivered when RF EMG signal amplitude remained stable around the target level. Once the tSCS was delivered, the participants stopped the muscle contraction and relaxed. Before conducting the contraction tasks, unconditioned spinal reflexes were obtained five times in a resting state. Subsequently, conditioned spinal reflexes were obtained five times during each task.

The calculated normalized conditioned reflex amplitudes were compared between the Sprinter and Control groups by using an unpaired t-test with Bonferroni correction. The root mean square value of the EMG signals in RF and BF immediately before tSCS (50-ms window) was denoted as background muscle activity (BGA) and was also compared between the two groups using an unpaired t-test with Bonferroni correction.

### Experiment 4: reciprocal inhibition induced by muscle vibration over RF

Mechanical vibration of RF muscle using a vibrator (B09Q7XM1K4, Topersun) was used to induce reciprocal inhibition of BF muscle. The vibration frequency was set at 30-Hz, because our previous study confirmed that a 30-Hz vibration frequency was sufficient to induce reciprocal inhibition of thigh muscles^30^. A vibrator was placed on the skin over RF (proximal side) using stands with movable arms (C-stand; Avenger, Cassola, Italy) to avoid changing the skin load. Before vibration, unconditioned spinal reflexes were obtained five times without vibration. For the measurements without vibration, the vibrator was attached to the skin with the vibration turned off. The vibrator was then turned on, and spinal reflexes were acquired five times during the vibration (i.e., conditioned reflex). tSCS was delivered 4–5 s after the vibration onset.

The calculated normalized conditioned reflex amplitudes were compared between the Sprinter and Control groups using an unpaired t-test. The root mean square value of the EMG signals in RF and BF immediately before tSCS (50-ms window) was denoted as BGA and was also compared between the groups using an unpaired t-test with Bonferroni correction. The level of significance was set at *p* < 0.05 in the Experiment 2–4.

## Supplementary Information

Below is the link to the electronic supplementary material.

Supplementary Material 1

Supplementary Material 2

Supplementary Material 3

## Data Availability

The datasets generated and analyzed during the current study are available as Supplementary Material.
