# Supplementary material for: Specific recruitment properties of spinal reflex of thigh muscle in sprinter
Source: Sci Rep. 2025 Nov 4;15:38641. doi: 10.1038/s41598-025-22504-2 (PMC12586427; doi:10.1038/s41598-025-22504-2)

Supplementary Material.

Individual recruitment traces for all participants

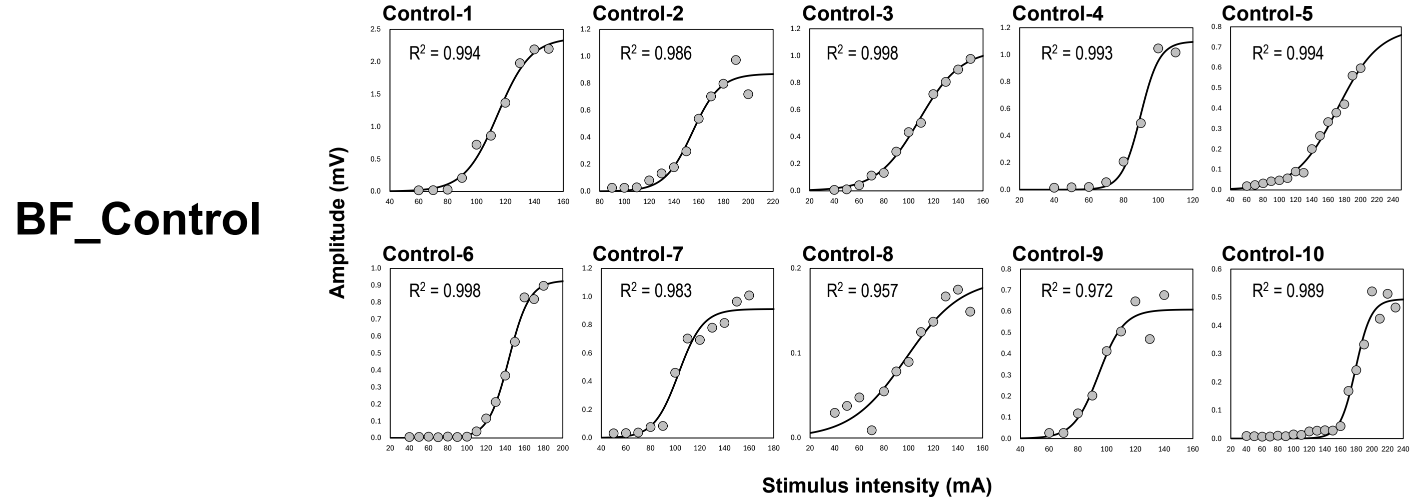

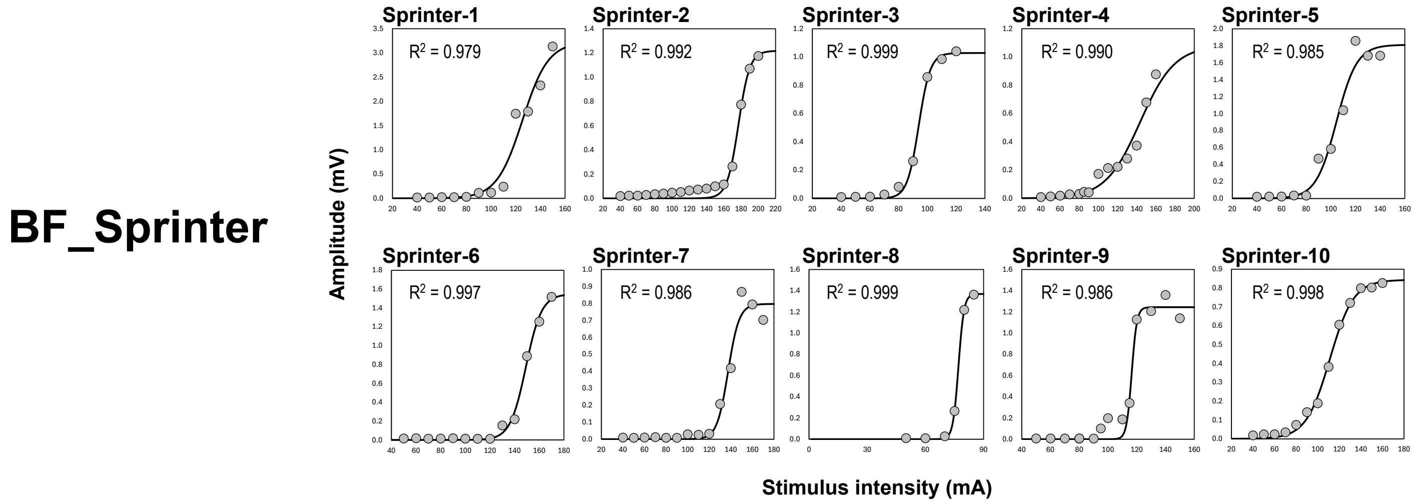

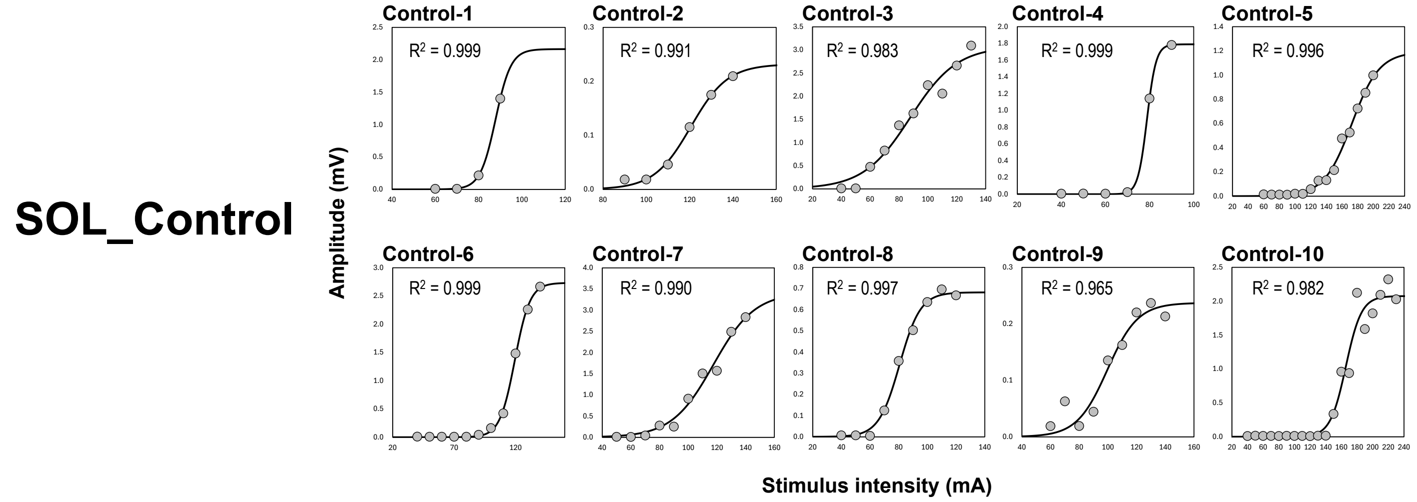

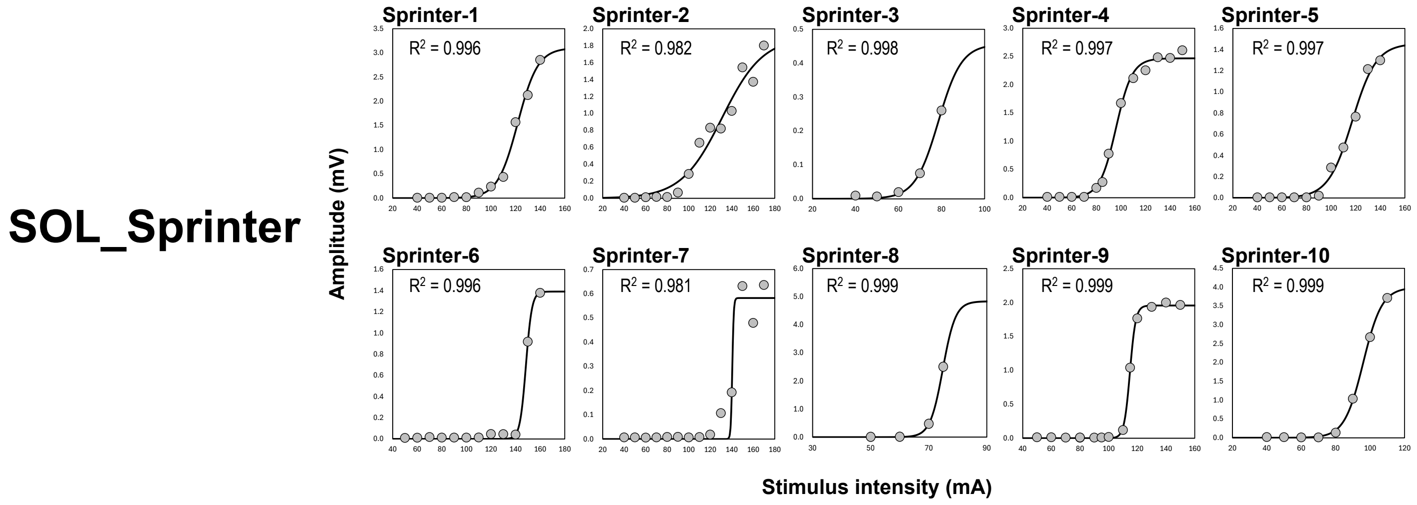

Supplement: Supplementary file 2 — Supplementary Material 2 [file 41598_2025_22504_MOESM2_ESM.pdf]
