# Supplementary material for: Specific recruitment properties of spinal reflex of thigh muscle in sprinter
Source: Sci Rep. 2025 Nov 4;15:38641. doi: 10.1038/s41598-025-22504-2 (PMC12586427; doi:10.1038/s41598-025-22504-2)
Supplement: Supplementary file 3 — Supplementary Material 3 [file 41598_2025_22504_MOESM3_ESM.pdf]

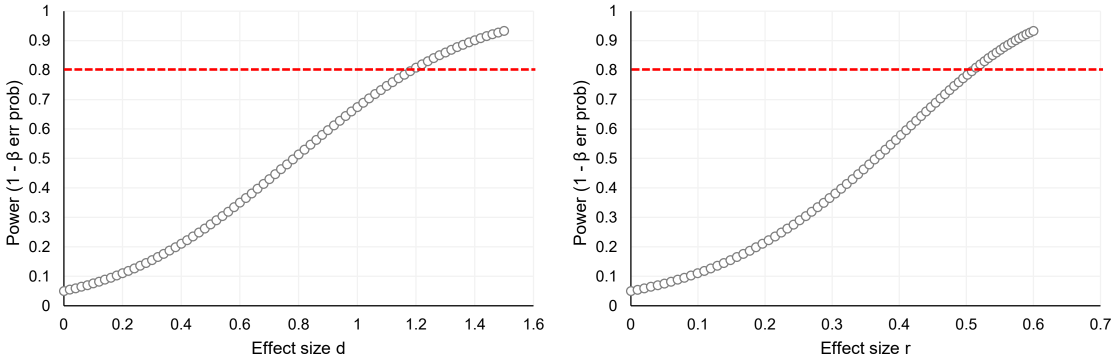

Sensitivity power curves (α = 0.05, N = 10 per group) showing detectable effect sizes for parametric (Cohen's *d*) and non-parametric (*r*) tests.

This figure displays sensitivity curves generated using G*Power, depicting the relationship between statistical power (1−β) and effect sizes in a two-group comparison. The left panel illustrates the parametric effect size (Cohen's *d*), typically used in t-tests, while the right panel shows the non-parametric effect size (*r*), used in Mann–Whitney U tests. The red dashed line indicates the conventional threshold of 80% power. With the current sample size (N = 10 per group), an effect size of approximately *d* = 1.1 or *r* = 0.5 is required to achieve 80% statistical power. These curves clarify the detectable effect size range for each test given the current design.
